# Prevalence of Musculoskeletal Pain and Analgesic Treatment Among Home-Dwelling Older Adults: Changes 1999–2019

**DOI:** 10.1093/geroni/igab046.3219

**Published:** 2021-12-17

**Authors:** Tuuli Lehti, Kaisu Pitkälä

**Affiliations:** University of Helsinki, Helsinki, Uusimaa, Finland

## Abstract

Pain has been shown to be undertreated in the older population. At the same time, the increased opioid use is of concern in the Western world. This study analyzes temporal trends in pain management among home-dwelling people aged 75 to 95 using cross-sectional cohort data spanning 20 years. The Helsinki Aging Study recruited random samples aged 75, 80, 85, 90, and 95 in 1999, 2009, and 2019. In total, 5,707 community-dwelling people participated in the questionnaire survey. Participants reported their medical diagnoses, regular prescription medications, and the presence of back pain or joint pain within the last 2 weeks (never, sometimes, or daily). We compared analgesics use in people reporting musculoskeletal pain and in people not reporting pain in 1999, 2009, and 2019. Of participants, 57–61% reported intermittent or daily musculoskeletal pain. The percentage of people taking a daily analgesic increased from 9% in 1999 to 16% in 2019. The use of NSAIDs decreased from 1999 to 2019, while the use of paracetamol increased from 2% to 11%. Of participants, 3% took daily opioids in 2019. Of those reporting daily musculoskeletal pain, 20% in 1999, 35% in 2009 and 32% in 2019 took regular pain medication. Pain remains undertreated in the older population, although the use of regular prescribed analgesics increased from 1999. The use of NSAIDs diminished, while the use of paracetamol increased. Daily opioid use remained modest from 1999 to 2019.

